# Multimode Fiber Specklegram Sensor for Multi-Position Loads Recognition Using Traversal Occlusion

**DOI:** 10.3390/s25061737

**Published:** 2025-03-11

**Authors:** Bohao Shen, Jianzhi Li, Zhe Ji

**Affiliations:** 1 School of Mechanical Engineering, Shijiazhuang Tiedao University, Shijiazhuang 050043, China; shenbohao0105@163.com (B.S.); jiyouyou1001@126.com (Z.J.); 2 Key Laboratory of Structural Health Monitoring and Control, Shijiazhuang Tiedao University, Shijiazhuang 050043, China

**Keywords:** multimode fiber specklegram, multiple perturbation, multi-position load, distributed sensor, traversal occlusion, shallow CNN, overfitting

## Abstract

Since an MMF-based distributed sensor requires the simultaneous measurement of multiple perturbation positions and their intensities, the collection of a large amount of specklegram data is time consuming and challenging for recognizing multiple perturbations. To address this issue, we propose a novel approach to recognize multi-position load using an MMF specklegram sensor, supported by theoretical analysis and experimental verification. Our study introduces a construction method for a multi-variable, multi-class, one-shot specklegram dataset, significantly enhancing the sample diversity for more perturbation positions and intensities in an MMF-distributed sensor recognition model. We theoretically derive the mathematical model of total local intensity for each region and investigate its sensitivity to the external perturbations. Based on these theoretical analyses, this paper proposes a specklegram traversal occlusion data augmentation with a shallow convolutional neural network (CNN) model to mitigate overfitting in specklegram datasets. Experimental validation using a multi-position load-recognition MMF demonstrates that our approach achieves nearly 100% accuracy in simultaneously recognized load positions and its magnitudes across up to 1545 distinct load forms. Furthermore, the shallow CNN model exhibits superior training efficiency and stability compared with the existing MMF sensing models. This work provides a proof of concept of a distributed sensor based on an MMF specklegram sensor, highlighting its potential for high-resolution distributed measurements under the diverse external perturbations. Our method represents a significant advancement in this field, offering a cost-effective and efficient solution for distributed sensing applications.

## 1. Introduction

In recent years, a single-mode fiber (SMF) distributed sensor has attained an exceptional resolution and long-distance capabilities. However, the inherent complexity of optical paths necessitates the use of expensive and bulky demodulation equipment [1,2]. In contrast, multimode fiber specklegram sensor systems offer a compact and cost-effective alternative, requiring only a photodetector and basic computation [3]. Meanwhile, multimode fiber specklegram sensors exhibit a high sensitivity to external perturbations, enabling applications in sensing, imaging, and communications [4,5,6]. However, an MMF specklegram sensor measures the perturbation by integrating all the small contributions at the different locations, which makes it much more complicated to locate each specific perturbation and measure their magnitudes. Despite these advantages, the challenge of simultaneously measuring multiple perturbation positions and their magnitudes remains a significant bottleneck in the development of MMF-based distributed sensors.

Researchers have developed vertical displacement sensors based on MMF specklegrams using algorithms such as the normalized intensity inner product (NIPC) [7,8,9,10] and gray level co-occurrence matrix (GLCM) [11]. In contrast, neural networks excel at decoupling specklegram information, enabling recognition of complex perturbations. For example, neural networks have been applied to the measurement of displacement [12,13,14,15], tip deflection [16], and torsion [17], achieving higher accuracy and better decoupling compared with NIPC and GLCM-based approaches. However, they only recognized single-parameter perturbations (magnitude or location), instead of multi-parameter perturbations.

On the other hand, deep learning has been applied to recognize position of perturbation due to its ability to extract specklegram features. For example, Cuevas et al. [18] localized three perturbations with 99% accuracy and ten with 71%, while these perturbations on each position are the exact same. Ding et al. [19] achieved tactile localization across nine positions, and Wei et al. [12] recognized steps with 92.83% accuracy over ten locations using ring-core fibers. Despite these advances, existing methods primarily localize perturbations but fail to simultaneously measure their magnitudes. Nevertheless, some researchers have advanced multi-position perturbation recognition, addressing both localization and intensity. For example, Sun et al. [20] proposed a two-axis displacement sensor, and Fujiwara et al. [21] proposed a multi-point curvature measurement method based on zero mean normalized cross-correlation coefficient (ZNCC) and realized small-angle bending recognition at two points. Similarly, Lu et al. [22] utilized neural network to recognize 55 bending combinations at up to three positions along an MMF with 93.5% accuracy. Although both studies demonstrated multi-variable recognition, there is some perturbation in their work (25 [21], 40 [20], and 55 [22]) and recognition error occurred, which cannot be afforded the re-equipment requirements of an MMF-distributed sensor.

This paper provides a proof-of-concept MMF specklegram-distributed sensor using traversal occlusion. A novel approach to rapidly construct a dataset with more perturbations is proposed, which enables a quick collection of large datasets with multi-perturbation intensities and positions. Each sample uniquely maps to a label, commonly referred to as one-shot specklegram dataset [23,24], which is beneficial to recognize many perturbations for the MMF-distributed sensors. Meanwhile, an experimental setup was designed where the multi-position loads with varying magnitudes were applied at the different positions along the fiber to validate multi-position perturbation recognition of FSSs.

The experimental results show the proposed model achieved nearly 100% accuracy in recognizing both load magnitudes and positions across multiple datasets. Our work extends the MMF sensing approaches by enabling simultaneous recognition of both position and magnitude across up to nine positions and 1545 unique load combinations. This represents an approximately fifteen times increase in perturbation complexity (1545 vs. 55 combinations in [22]). The performance of sensor between our work and existing methods is shown in Table 1.

## 2. Theoretical Analysis

### 2.1. Statistical Analysis of Specklegram Variations Induced by Perturbations

To study the local response of a specklegram under distinct perturbations, this paper theoretically derived an MMF-coupling mode equation and analyzed the effects of perturbations on a specklegram and the key factors driving specklegram variations and regional differences.

In MMF, mode coupling can be considered as the interaction of optical fields when propagating through the fiber. The resulting complex field distribution at the MMF output is the superposition of the complex amplitudes of all propagating modes and can be expressed as [25]

(1) $${\tilde{E}}_{\mathrm{out}}(x,y)=\sum_{p}^{N}a_{p}(L)e_{p}(x,y)$$

where N represents the number of propagating modes in the MMF, L represents the fiber length, and $a_{p}$ and $e_{p}$ are the amplitude and transverse electric field distribution of the *p*-th mode at the output end, respectively.

Perturbations in MMF arise from geometric deformations (e.g., micro bending) or refractive index fluctuations. These perturbations induce random variations in coupling coefficients $C_{pq}$, which denotes the interactions of the different modes. The coupled-mode formulation for a uniform structure with a perturbation within or near its boundaries can be described by [26]

(2) $$\frac{da_{p}(z)}{dz}+i\beta_{p}a_{p}(z)=\sum_{q}^{N}C_{pq}(z)a_{q}(z)$$

where $a_{p}(z)$ and $a_{q}(z)$ represent the amplitude of *p*-th mode and *q*-th mode along the propagation distance z, respectively. $\beta_{p}$ is the *p*-th modal propagation constant.

The coupling coefficients $C_{pq}$ can be expressed as [26]

(3) $$C_{pq}=\frac{\omega }{2}\int_{A_{\infty }}\left[\Delta \varepsilon e_{p}\cdot e_{q}^{*}\right]dA$$

where ω represents the angular frequency, Δε represents the changes in dielectric constant, A represents the cross section of MMF. Commonly, transverse electric field distribution $e_{p}$ is the eigen solution of the original wave equation without disturbance. Therefore, the perturbations mainly result in the dielectric constant change, and further the coupling coefficient $C_{pq}$.

Under random coupling, the mode amplitudes $a_{p}(z)$ are random variables, whose statistical properties depend on the statistical characteristics of $C_{pq}$. The coherent superposition of modes generates a specklegram with statistical intensity distributions. Thus, analyzing the intensity distribution offers insight into the perturbations. The specklegram intensity at the output is given by [27]

(4) $$I_{\mathrm{out}}(x,y)={\left|{\tilde{E}}_{\mathrm{out}}(x,y)\right|}^{2}={\left|\sum_{p}^{N}a_{p}(L)e_{p}(x,y)\right|}^{2}$$

where $a_{p}(L)$ is calculated by transfer coefficient $R_{pq}$ coupled by p and q which presents the order of mode

(5) $$a_{p}(L)=\sum_{q}^{N}R_{pq}a_{q}(0)$$

Equations (2) and (5) can be written in matrix form as

(6) $$\frac{da(z)}{dz}+iBa(z)=C(z)a(z)$$

(7) a(L)=R(L)a(0)

(8) $$a(z)={\left[a_{1}(z),a_{2}(z),\ldots ,a_{N}(z)\right]}^{T}$$

(9) $$B=\mathrm{diag}(\beta_{1},\beta_{2},\ldots ,\beta_{N})$$

where C(z) is the random coupling matrix, $\beta_{n}$ is the mode propagation constant, R(L) is the N×N propagation matrix.

The influencing factors of each region on the specklegram were further analyzed. Assuming that the output specklegram is divided into a 3 × 3 grid, where each tile represents a local region, denoted as $A_{i}$ (*i* is the index of the subgraph, *i* ∈ [1, 9]), the features of the specklegram within each tile $A_{ij}$ can be characterized by the local light intensity distribution, and the total local intensity for each region can be expressed as

(10) $$\begin{array}{ll} I_{i} & =\int_{A_{i}}I_{\mathrm{out}}(x,y)dxdy\\ & =\int_{A_{i}}{\left|\sum_{p}^{N}a_{p}(L)e_{p}(x,y)\right|}^{2}dxdy\\ & =\sum_{p}^{N}{\left|a_{p}(L)\right|}^{2}\int_{A_{i}}{\left|e_{p}(x,y)\right|}^{2}dxdy\\ & +\sum_{p\neq q}^{N}a_{p}(L)a_{q}^{*}(L)\int_{A_{i}}e_{p}(x,y)e_{q}^{*}(x,y)dxdy \end{array}$$

According to the different coupling modes, Equation (10) is divided into self-interference and cross-interference:

(11) $$I_{i}^{\mathrm{self}}=\sum_{p}^{N}{\left|a_{p}(L)\right|}^{2}\int_{A_{i}}{\left|e_{p}(x,y)\right|}^{2}dxdy$$

(12) $$I_{i}^{\mathrm{cross}}=\sum_{p\neq q}^{N}a_{p}(L)a_{q}^{*}(L)\int_{A_{i}}e_{p}(x,y)e_{q}^{*}(x,y)dxdy$$

Therefore, the total intensity of the tile can be written as

(13) $$I_{i}=I_{i}^{\mathrm{self}}+I_{i}^{\mathrm{cross}}$$

Self-interference $I_{i}^{\mathrm{self}}$ reflects the contribution of a single mode, which primarily depends on the spatial distribution of the mode field. In contrast, the cross-interference $I_{i}^{\mathrm{cross}}$ represents the interference contribution of the different modes. Based on Equations (2) and (3), perturbations result in the changes of dielectric constant Δε and coupling coefficient $C_{pq}$ of each mode, which in turn alter the amplitude of the p-th mode $a_{p}$. In addition, the total local intensity for each region depends on $a_{p}$. Then, the statistical distribution of changes is uniform and does not have regional differences. Based on Equations (10) and (13), the total local intensity for each region also depends on the transverse electric field distribution of the *p*-th mode $e_{p}$; its variations consist of two parts, a single mode and the interference of the different modes. Hence, the variation of speckle patterns in different regions under disturbance has regional differences due to the different lateral electric field distribution between each of the modes. Therefore, the key influencing factor of each region in specklegram under external perturbations is the lateral distribution of each mode, which dictates the relative contribution of modes to the intensity in each tile. Especially for higher-order modes, they are usually more sensitive to perturbations [28].

Higher-order modes exhibit a greater sensitivity to external perturbations compared to lower-order modes due to their larger spatial overlap with fiber cladding and stronger evanescent field interactions. This distinction arises because higher-order modes propagate closer to the core-cladding interface, where mechanical deformations (e.g., bending, compression) disproportionately alter their phase and intensity. In contrast, lower-order modes remain largely confined to the fiber core, resulting in weaker perturbation coupling.

Perturbations induce stronger mode coupling for higher-order modes, amplifying specklegram variations in regions where these modes dominate (typically peripheral region in specklegram). Consequently, edge and corner regions of the specklegram show greater sensitivity. This spatially non-uniform sensitivity indicates the differences in disturbance characteristics across different local regions of the speckle pattern.

### 2.2. Analysis of Specklegram Variation Under Perturbation

**Figure 1:**
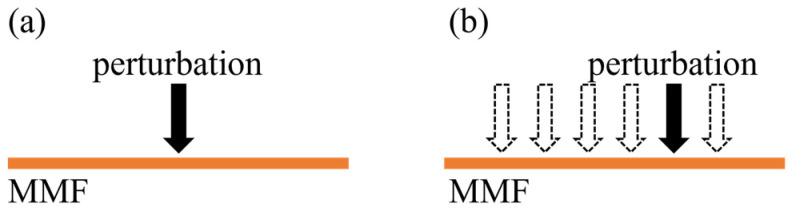
Schematic diagram of experimental setup for studying specklegram variations in MMF: (**a**) various perturbation intensities applied at the same position; (**b**) same perturbation intensity applied on different positions (dotted arrows indicate perturbation locations).

Two experiments were conducted on MMFs (one-meter step-index MMF with a core diameter of 50 μm, a numerical aperture of 0.22) to study the spatial differences in specklegram variations: the first was applied various perturbation intensity at the same point, while the second one applied same perturbation intensity on the different positions. The experimental schematic diagram is shown in Figure 1.

**Figure 2:**
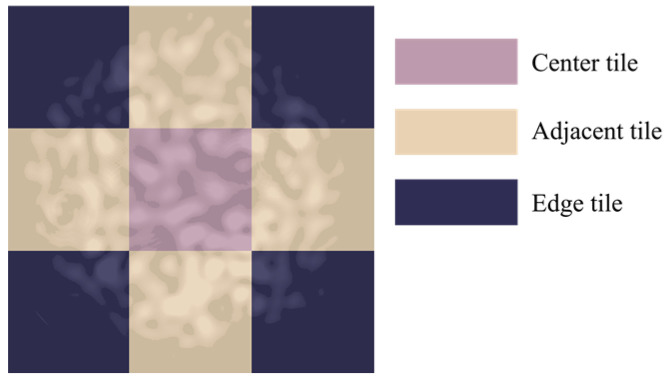
Schematic diagram of the 3 × 3 division of specklegram images.

For each condition, ten groups were tested, with ten specklegrams captured per group. Each image was divided into nine tiles in a 3 × 3 grid, categorized as center, adjacent, or edge tiles, as shown in Figure 2.

**Figure 3:**
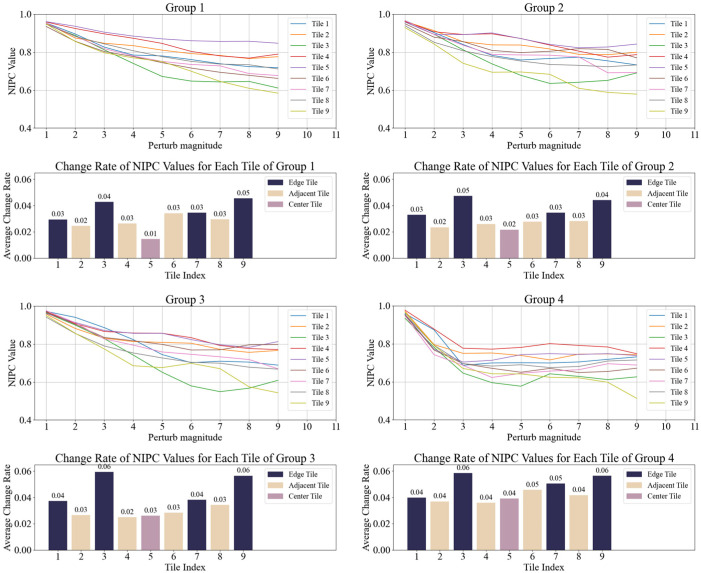
NIPC and average change rate of each tile under varying perturbation intensities on same position.

**Figure 4:**
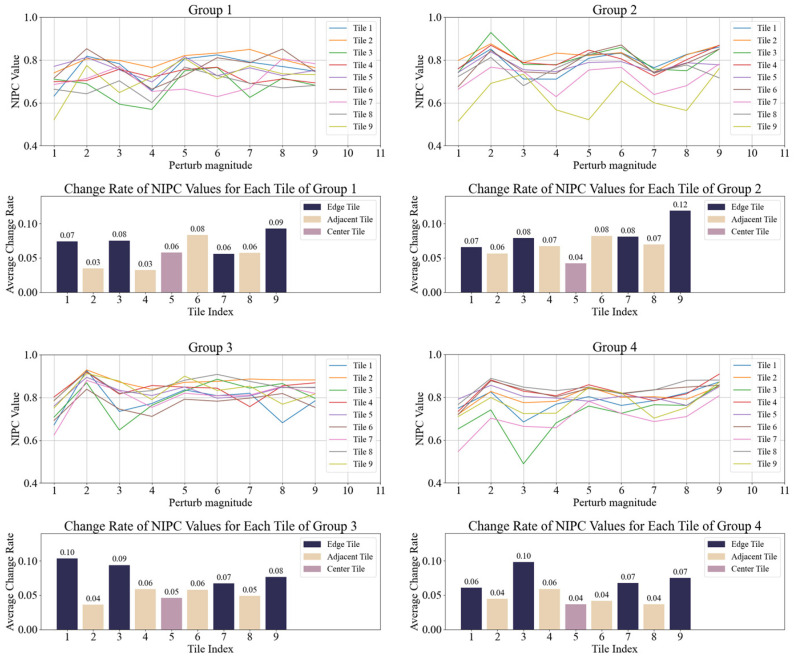
NIPC and average change rate of each tile under perturbation at different positions.

**Figure 5:**
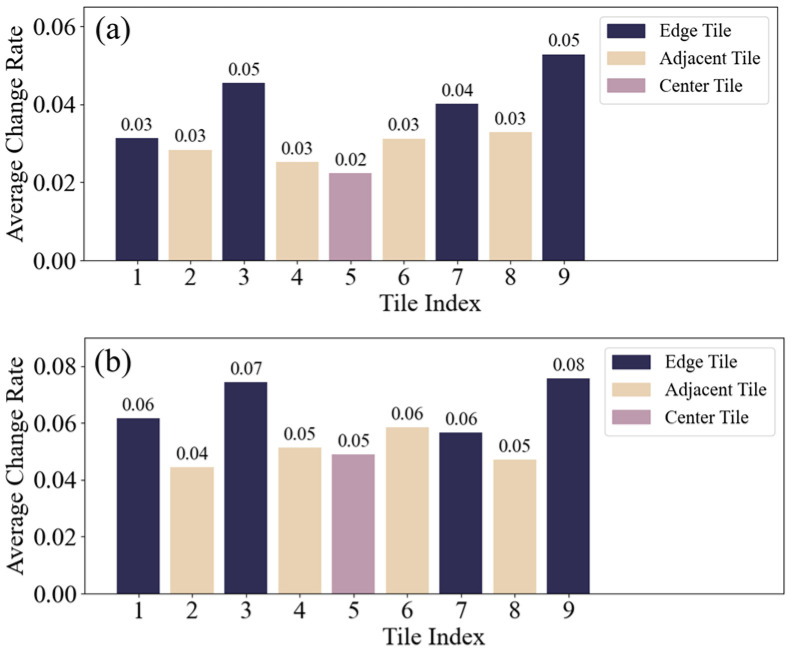
Average change rate of ten group for each tile: (**a**) continuous varying perturbation intensities on same position; (**b**) same perturbation at different positions.

The first specklegram in each group was used as a reference, and NIPC values were calculated for each tile in the remaining nine specklegrams. The NIPC values and average rate of change were then computed for all tiles. Results of four groups for varying perturbation intensities are shown in Figure 3, and for perturbations of different intensities in Figure 4. Then, the average rate of change for each tile in all ten groups was calculated, as illustrated in Figure 5.

As shown in Figure 3, Figure 4 and Figure 5, the NIPC values gradually decrease with the continuous perturbation intensities. However, when the same perturbation was applied at the different positions, we find that the NIPC values fluctuate with perturbation location. Nevertheless, the central region of the specklegram exhibits smaller variation amplitudes, while the adjacent and edge regions show more significant changes. Then, each region of specklegram encodes distinct information of perturbation, forming a statistically localized mapping relationship, which is consistent with the theoretical analysis.

## 3. Method

### 3.1. Specklegrams Acquisition and Datasets Construction

To train a neural network model for multi-position perturbation recognition in MMF, this work established an experimental framework by applying loads of varying magnitudes at the different positions along the MMF.

The experimental setup included a laser source, an MMF, and a high-resolution CCD camera to capture the specklegram. Perturbations were applied by randomly placing discrete weights along the fiber, and the resulting specklegram images were recorded systematically. The components of the experimental setup are detailed as follows:

(1) Light source: A laser emitting at a wavelength of 530 nm was coupled into the MMF via an SMA905 interface. The laser is operated using an adjustable power level, and a power setting of 25 mW selected to achieve clear and high-contrast specklegram images.
(2) Multimode fiber: A 1 m step-index MMF with a core diameter of 50 μm and a numerical aperture (NA) of 0.2 was utilized. A collimator was employed to adjust the size of the output specklegram.
(3) Detection system: Specklegrams at the output end of the MMF were captured using a high-resolution CCD camera. The camera had a pixel size of 1.85 μm, a resolution of 4032 × 3037 pixels, and a photosensitive area of 7.4 mm × 5.6 mm. To prevent overexposure, an attenuator was used to reduce the laser power to a suitable level for image acquisition. The CCD camera is a laser beam quality analyzer manufactured by Wavelab Technology.

**Figure 6:**
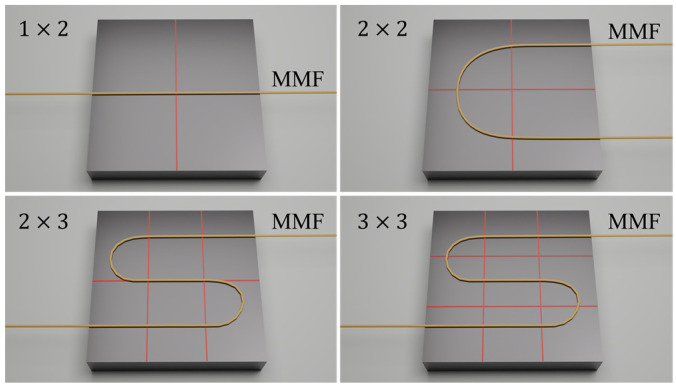
Diagram of grid divisions and fiber placement.

**Figure 7:**
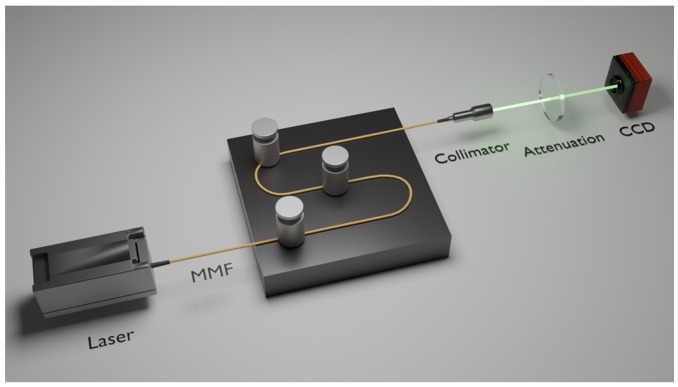
Diagram of loads on MMF (the condition of three loads applied on the 3 × 3 plane).

For the experimental setup, the MMF was fixed onto a flat plane, which was divided into multiple cells using four grid configurations: 1 × 2, 2 × 2, 2 × 3, and 3 × 3, as illustrated in Figure 6. Weights were randomly placed within these cells, and each weight was directly placed on the fiber in its respective cells. Figure 7 shows one of the applied loads in the experiment three weights are randomly placed on a 3 × 3 grid.

Based on the placement and quantity of weights, various combinations can be formed. The total number of combinations $N_{\mathrm{combination}}$ is calculated as

(14) $$N_{\mathrm{combination}}=\sum_{k=0}^{i}\frac{(m\cdot n)!}{k!\left(m\cdot n-k\right)!}\cdot j^{k}$$

where m and n represent the number of rows and columns in the grid, respectively. i represents the maximum number of load positions applied across the grid, and j represents the maximum number of weights that can be applied per cell.

**Figure 8:**
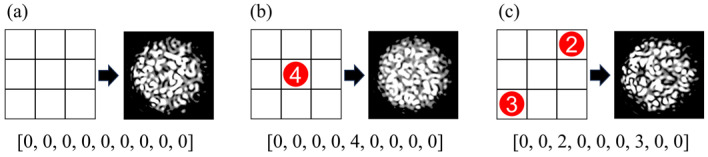
Diagram of perturbation position and intensity with the corresponding specklegram sample annotation: (**a**) [0, 0, 0, 0, 0, 0, 0, 0, 0] indicates no weights are applied; (**b**) [0, 0, 0, 0, 4, 0, 0, 0, 0] indicates four weights are applied in the fifth position on the planar; (**c**) [0, 0, 2, 0, 0, 0, 3, 0, 0] indicates two and three weights are applied in the third and seventh position on the planar, respectively.

For instance, when weights were randomly applied to up to two cells on a 3 × 3 grid, with a maximum load of 30 g per cell, a total of 1351 unique combinations were formed ($\sum_{k=0}^{2}\frac{(3\cdot 3)!}{k!\left(3\cdot 3-k\right)!}\cdot 6^{k}=1351$). Then, a dataset of 1351 specklegram categories was constructed. Each annotation of sample consists of nine values representing the 3 × 3 grid areas, where 0 indicates no weight and other numbers denote the amount of 5 g weights applied, as shown in Figure 8. As indicated by the sample annotation, the experimental objective is to achieve multi-variable, multi-class recognition.

Similarly, based on Equation (14), Table 2 summarizes the load combinations for grids configured as 1 × 2, 2 × 2, 2 × 3, and 3 × 3 on the plane. Given the manual efficiency of placing weights and collecting specklegram images at approximately 300 images per hour, the dataset size was approximately 1500 specklegram categories in a collection time of about 5 h.

### 3.2. Implementation Framework

**Figure 9:**
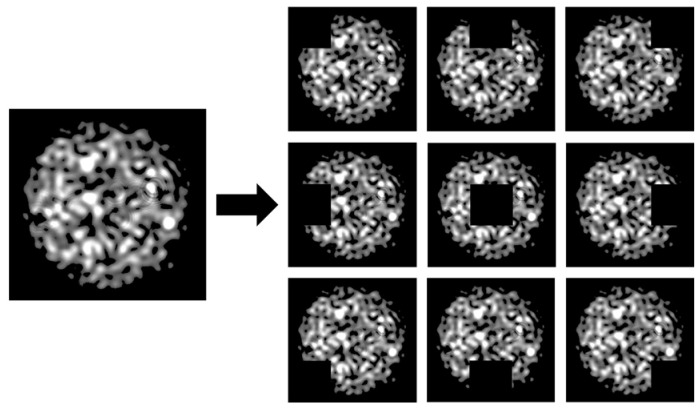
Generation of specklegram by traversal occlusion.

This study introduces a specklegram enhancement strategy of traversal occlusion that forces the model to focus on both global and local features of the specklegram, which alleviates overfitting. The input image was segmented into a 3 × 3 grid, producing nine equally sized tiles. Each tile was selected for occlusion, with its pixel values replaced by a black patch to mask the visual information in that region. Figure 9 illustrates the generation of specklegram by traversal occlusion, where one of the nine tiles in the specklegram is occluded. Hence, the dataset size is increased.

Mathematically, for an image I of size h×w, we divide I into tiles using the following coordinates:

(15) $${\mathrm{region}}_{i,j}=I\left(\frac{i\cdot h}{3}\leq y<\frac{(i+1)\cdot h}{3},\frac{i\cdot w}{3}\leq x<\frac{(i+1)\cdot w}{3}\right)$$

where *i*,*j* ∈ {0,1,2} denote the row and column indices of the grid, respectively. For each selected tile, all pixel values within that tile are set to zero, effectively blacking out the area, which generates partially occluded versions of the original image. The dataset was expanded tenfold by this method. Accordingly, the model ability is enhanced to recognize robust features under varying external perturbations.

We developed a custom dataset loader that dynamically applies occlusion augmentation during runtime. For each image, the loader traversal selects tiles to occlude and then applies preprocessing steps, including resizing, grayscale conversion, and normalization. The process is shown as Algorithm 1.
**Algorithm 1** Augmented MMF specklegrams preprocessing with traversal occlusion**Inputs:** Dataset directory *D*, input dimension *d***For each:** specklegram *I* ∈ *D*: 1. Divide I into a 3 × 3 grid of tiles 2. Iteratively select one tile from the grid 3. For each selected tile, set all the pixel values to zero 4. Resize *I* to a fixed dimension *d* × *d* 5. Convert *I* to grayscale 6. Apply normalization:$I\leftarrow \frac{I-\mu }{\sigma },\;where\;\mu =0.5,\;\sigma =0.5$ 7. Store processed image *I* and associated label *y***Output:** Processed dataset and labels (*X*, *Y*)

Furthermore, to achieve fast and stable training, a shallow convolutional neural network was developed. Unlike deep models such as VGG16 [29] and ResNet-18 [30], which employ hierarchical convolutional layers to extract progressively abstract features, the proposed shallow CNN prioritizes global spatial correlations in specklegrams. This design aligns with the physical nature of multimode interference, where perturbations induce distributed intensity changes across the specklegram. The reduced depth and parameter count (1 M in shallow CNN vs. 138.36 M in VGG16) minimize overfitting risks while maintaining high training efficiency. The proposed shallow CNN architecture consists of two convolutional layers, each followed by a max-pooling operation. The first convolutional layer utilized eight filters with a kernel size of 3 × 3, while the second convolutional layer had sixteen filters with the same kernel size. Both convolutional layers incorporated a rectified linear unit (ReLU) activation function to introduce non-linearity. Max-pooling was applied with a pooling size of 2 × 2, halving the spatial dimensions of the feature maps after each pooling operation.

**Figure 10:**
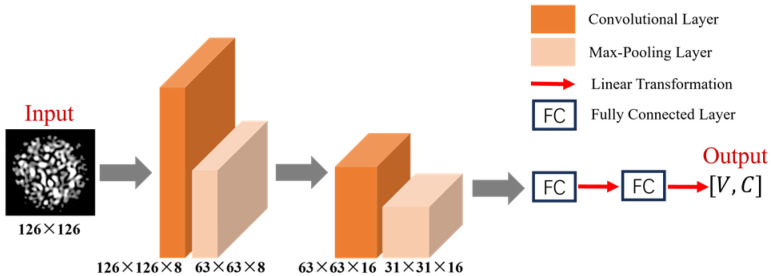
Architecture of the shallow CNN.

Following the convolutional layers, the feature maps were flattened into a one-dimensional vector and passed through two fully connected layers. The final fully connected layer outputs a tensor of shape [V,C], where V represents the number of variables, and C is the number of possible categories for each variable. This structure enabled the network to predict C categories for each of the V variables, with the interpretation that a maximum load of C per cell is applied at V positions. Minimizing computational complexity using a shallow neural network could reduce overfitting and facilitate fast training. The network structure is illustrated in Figure 10.

### 3.3. Training and Evaluation

The training process was carried out using the Adam optimizer with a learning rate of 1 × 10^−5^, which provided an adaptive learning rate for each parameter in the model. The loss function employed was the sum of cross-entropy losses across the nine variable categories in Equation (16). Specifically, for each image, a separate cross-entropy loss is calculated for each of the nine variables, and the overall loss is calculated as the sum of these individual losses in Equation (17). Hence, the model learned to recognize all nine variables simultaneously.

(16) $$L_{\mathrm{CrossEntropy}}=-\sum_{c=1}^{C}y_{c}\log\left({\hat{y}}_{c}\right)$$

(17) $$L_{\mathrm{Sample}}=\sum_{i=1}^{V}\left(-\sum_{c=1}^{C}y_{c}\log\left({\hat{y}}_{c}\right)\right)$$

where C represents the number of categories for each variable, V represents the variables of the label, $y_{c}$ is the one-shot encoding of the true class (with 1 at the position of the true class and 0 elsewhere), ${\hat{y}}_{c}$ represents the predicted class probability distribution (obtained by SoftMax function).

The dataset was randomly split into training and testing subsets, with 80% of the data used for training and the remaining 20% used for evaluation. Training was performed over a total of 1000 epochs, with each epoch consisting of multiple iterations based on the size of the training dataset and the batch size of 128. During each iteration, the model weights were updated using backpropagation, where the gradient of the loss with respect to each parameter was computed. Accordingly, the optimizer then adjusted the weights, making the model progressively minimize the overall loss and improve its predictive performance.

A multi-variable, multi-class classification approach was adopted to recognize specklegrams. As shown in Figure 8, the labels are divided into several variables based on the number of perturbation positions, and the perturbation intensity at each location is further classified and recognized. Four classification metrics were used to evaluate the model during training:

(1) Exact match ratio (EMR): The exact match ratio is mentioned below which explains the percentage of instance whose recognized labels are exactly matching the same true set of labels. It can also be used to describe the recognition accuracy of the model.

(18) $$\mathrm{EMR}=\frac{1}{N}\sum_{i=1}^{N}1\left(y_{\mathrm{true},i}=y_{\mathrm{pred},i}\right)$$

where N represents the total number of samples, $y_{\mathrm{true},i}$ represents the true label of the *i*-th sample, $y_{\mathrm{pred},i}$ represents the predicted label of the *i*-th sample, the indicator function returns a value of 1 if all label positions match between $y_{\mathrm{true},i}$ and $y_{\mathrm{pred},i}$, and 0 otherwise.

(2) Per-label accuracy (PLA): This metric is used to evaluate the model performance by calculating the average accuracy across all variables of the label. Unlike EMR, PLA provides a more granular insight into the ability to predict individual variable of labels correctly, independent of the correctness of other labels. The PLA is expressed as

(19) $$\mathrm{PLA}=\frac{1}{N\cdot d}\sum_{i=1}^{N}\sum_{j=1}^{d}1\left(y_{\mathrm{true},i,j}=y_{\mathrm{pred},i,j}\right)$$

where d represents the total variables of the label, $y_{\mathrm{true},i,j}$ represents the true value of the *j*-th variables for the *i*-th sample, $y_{\mathrm{pred},i,j}$ represents the recognized value of the *j*-th variable for the *i*-th sample.

(3) F1 score: The F1 score is computed for each of the nine label variables, and their average was reported as the final metric. The F1 score is particularly valuable in scenarios involving imbalanced data, as it balances both precision and recall, offering a more comprehensive evaluation of the model performance. The F1 score can be calculated as

(20) $$F1=\frac{1}{N}\sum_{i=1}^{N}\frac{{\mathrm{Precision}}_{i}\cdot {\mathrm{Recall}}_{i}}{{\mathrm{Precision}}_{i}{+\mathrm{Recall}}_{i}}$$

where ${\mathrm{Precision}}_{i}$ and ${\mathrm{Recall}}_{i}$ are the precision and recall of the *i*-th sample, respectively. The F1 score effectively captures the harmonic means of precision and recall, ensuring that both metrics are equally weighted in evaluating the model ability to make accurate predictions.

(4) Hamming loss: In contrast to PLA, the Hamming loss equation computes the average of incorrect variable in a label of an instance. The lower the value, the higher the performance of the classifier, as it is a loss function.

(21) $$L_{\mathrm{Hamming}}=\frac{1}{N\cdot d}\sum_{i=1}^{N}\sum_{j=1}^{d}1\left(y_{\mathrm{true},i,j}\neq y_{\mathrm{pred},i,j}\right)$$

where the metric evaluates the proportion of mismatched perturbance in a position between the true and predicted labels.

## 4. Experiments and Results

### 4.1. Experimental Setup and Specklegrams Collection

To validate the proposed method and achieve multi-position load recognition using an MMF, experiments were conducted as the setups illustrated in Figure 6 and Figure 7. The MMF was laid flat in various configurations, and specklegram images were captured for each form of load. The datasets contained a large number of specklegram categories (at least 121 and up to 1545 unique load combinations), with each category represented by a single specklegram image, involving perturbations at multiple positions and varying magnitudes. To achieve the planar structure deformation under applied loads, plastic foam was chosen as the planar carrier. This material exhibits good deformation under applied load and reliably restores to its original shape after load removal, ensuring both the repeatability and reliability of the experiment.

**Figure 11:**
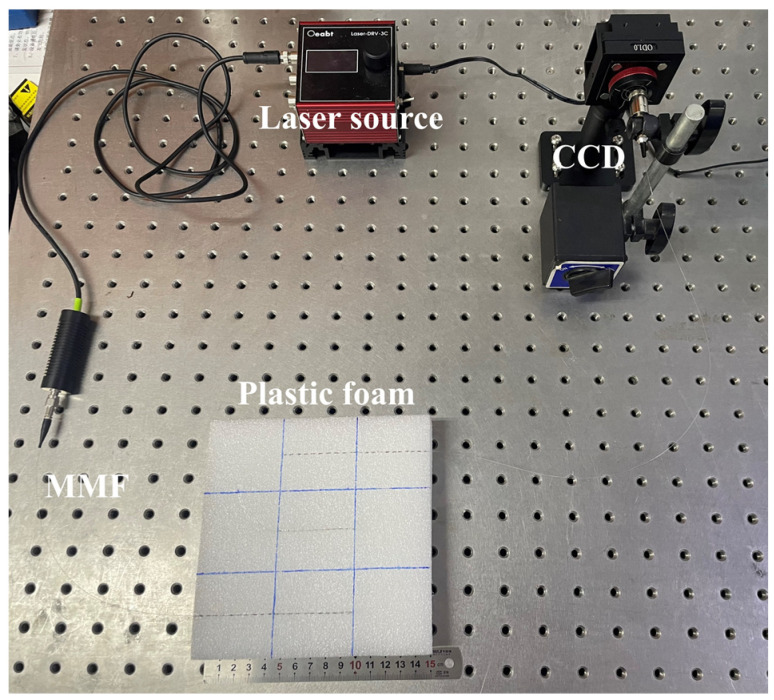
Diagram of the experimental setup for recognition of the load distribution on the foam.

**Figure 12:**
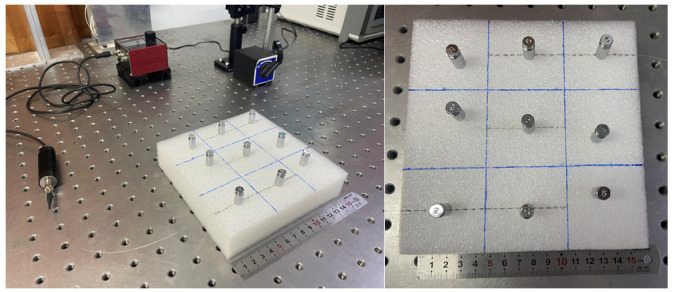
Diagram of the positions of the weight on the foam.

For instance, a foam was divided into 3 × 3 grid, and each has a side length of 5 cm. A laser source and a CCD detector were incorporated into the optical fiber system to record variations in the transmitted light intensity within the fiber, as shown in Figure 11. During the experiment, several loads were applied using a weight of 5 g at the center of each cell and place on the fiber to ensure consistency and repeatability of load. The positions of all weights that were placed at all cells are shown in Figure 12.

### 4.2. Loads Sensing with MMF Specklegrams

**Figure 13:**
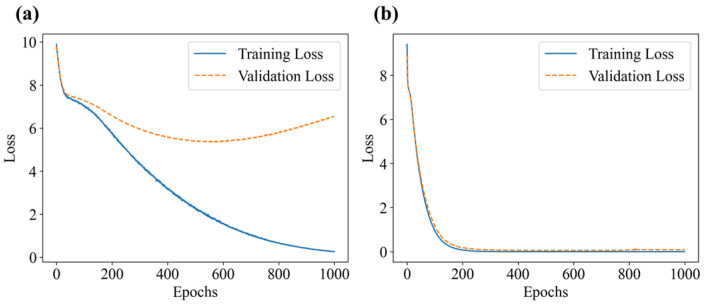
Loss function plot during the training process: (**a**) without occlusion; (**b**) with occlusion.

**Figure 14:**
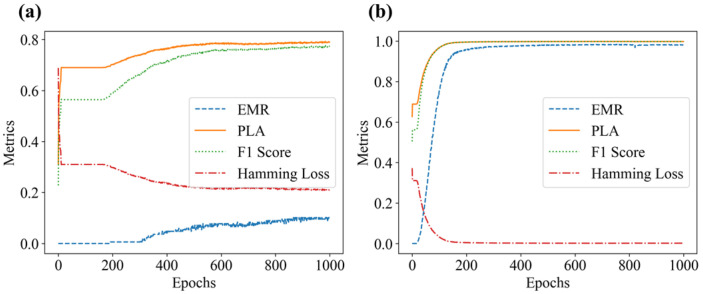
Model evaluation metrics plot: (**a**) without occlusion; (**b**) with occlusion.

According to Table 1, on the condition of the 3 × 3 grid, three perturbation positions, and at most two weights per cell. There are up to 835 possible load combinations. Specklegram corresponding to these combinations were captured, resulting in a one-shot dataset containing 835 specklegram images and 835 specklegram categories. The shallow CNN models were trained on this dataset both without and with the occlusion method applied, respectively. Those loss function and evaluation metrics during the training process are illustrated in Figure 13 and Figure 14, respectively. The results indicate that when the occlusion method was not applied, the validation loss converges more slowly and is not close to the training loss, suggesting that the model suffers from overfitting. In addition, the model without the occlusion resulted in a high PLA and yielded a low EMR, suggesting that the model could recognize intensity of perturbations at some location but not accurately recognize the whole distribution on the planar surface. When the occlusion method was applied, the validation loss closely aligned with the training loss over epochs, and the EMR consistently approached 1 (even reached 1). The F1 score of the model without occlusion is relatively lower compared with the model with occlusion whose F1 score approaches 1, indicating that the occlusion method enables the model to achieve better recognition ability. The Hamming loss without occlusion is relatively higher than one with occlusion, indicating that the occlusion traversal method enhances the model’s performance of recognition of specklegrams.

**Figure 15:**
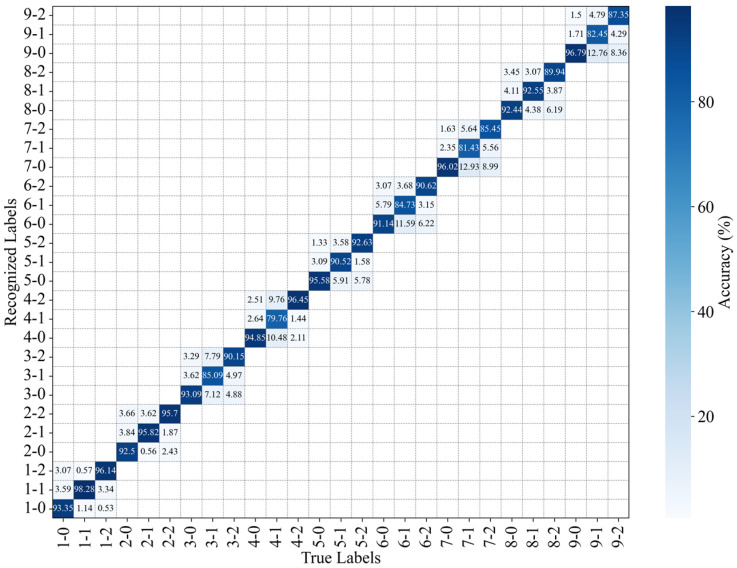
Recognition accuracy of the position and intensity of perturbations without occlusion.

**Figure 16:**
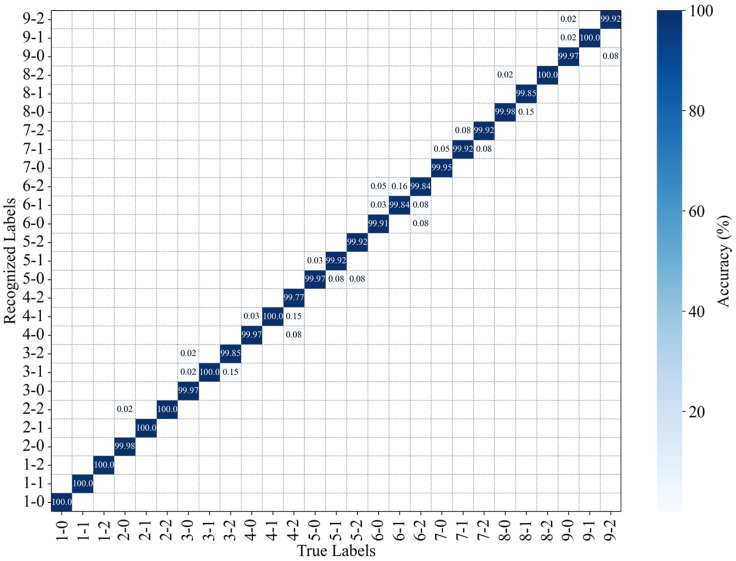
Recognition accuracy of the position and intensity of perturbations with occlusion.

The PLA of each position without occlusion and with traversal occlusion is shown in Figure 15 and Figure 16, respectively. The horizontal axis represents the position and magnitudes of the applied load, while the vertical axis represents the recognition results for the specklegram under the given load conditions. For example, the true label 3-1 indicates that one weight was applied at position 3. The corresponding vertical axis values, 3-0, 3-1, and 3-2, represent the recognition results under this loading: 85.09% of the speckle patterns were correctly recognized, 7.12% were incorrectly recognized as zero weight applied at position 3, and 7.79% were incorrectly recognized as two weights applied at position 3. It indicates that the model without occlusion has many recognition errors, while the method with occlusion is more accurate in recognizing the load magnitudes at each position. As previously analyzed, each tile of specklegram contains different pieces of perturbation information. Consequently, the model without occlusion learns excessive local features, and leading to overfitting, while the model with traversal occlusion effectively eliminates the overfitting issue by discarding the local speckle features.

**Figure 17:**
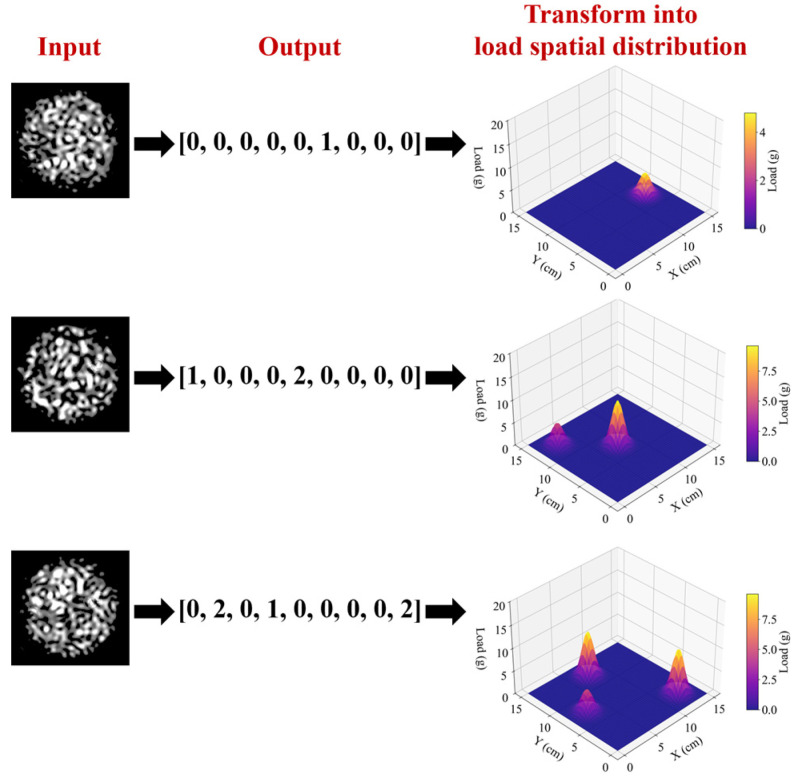
Process of converting input specklegram into load spatial distribution.

The spatial load distribution on the plane was obtained by inputting the specklegram image into the trained model, as illustrated in Figure 17. For a 15 cm × 15 cm planar surface, the model successfully recognized the number of weights corresponding to each grid position. Then the results were transformed into a 3D load distribution, providing a visual representation of load variations across the plane. The proposed method demonstrates an effective transformation of specklegrams into accurate planar load distribution.

**Figure 18:**
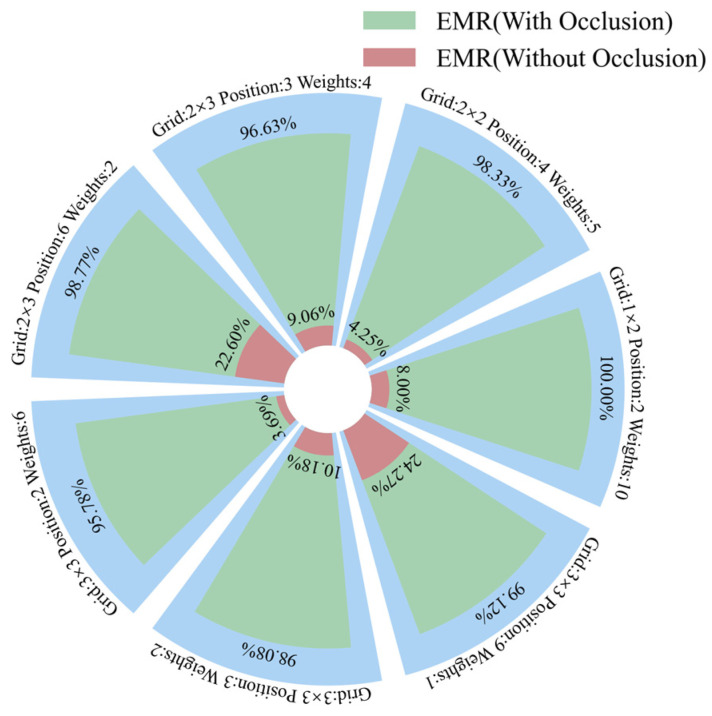
EMR of shallow CNN on seven datasets.

Based on all the combinations listed in Table 1, the proposed shallow CNNs without occlusion and with traversal occlusion were employed to recognize these combinations, with the results shown in Table 3 and Table 4, respectively. The EMRs of two methods on seven datasets are shown in Figure 18. The model without occlusion can only achieve a recognition accuracy up to 24.27%, while the one with occlusion can achieve a recognition accuracy of at least 95.78%, and even 100%. The F1 score of the model with traversal occlusion is close to 1, and the Hamming loss is close to 0, indicating that the model demonstrates excellent performance. These results demonstrate that the proposed method greatly improves the recognition accuracy of multi-position load on the MMF. The proposed traversal occlusion approach to improve the model’s specklegram recognition ability on multi-variable, multi-class, one-shot specklegram datasets is feasible. The main reason for recognition errors in some datasets might be attributed to the location error of manually placing these weights.

### 4.3. Comparation with Existing Models

The performance of the proposed method was compared with the other trained models without occlusion. Additionally, comparative analyses were conducted with ResNet-18 [17,19,20,30] and VGG16 [13,14,29]. The evaluation metrics include EMR to assess the accuracy of each specklegram recognition, F1 score to evaluate the model performance, and Hamming loss to measure the independent error rate of each position across all samples. The results are summarized in Table 5, Table 6, Table 7 and Table 8.

As shown in Table 5, Table 6, Table 7 and Table 8, the shallow CNN still achieved the highest accuracy of 24.27% among the model without occlusion, while the recognition accuracy improves significantly across various datasets, achieving a minimum of 96.63% as the model utilized traversal occlusion. The F1 score of the models with traversal occlusion is close to 1, and the Hamming loss is near 0, indicating that the models with traversal occlusion demonstrate a better performance than the models without occlusion. The experimental results indicate that the shallow CNN model achieves relatively high recognition accuracy even without utilizing the traversal occlusion, highlighting its strong anti-overfitting capability in specklegram recognition. When the traversal occlusion method is applied, all models achieve nearly 100% EMR, indicating that the proposed method effectively extracts the multiple perturbation locations and intensities from the global features of specklegrams. These results demonstrate that the method achieves high-precision decoupling of specklegram images. In addition, these results confirm that, despite the limited dataset size and complex perturbations, the proposed method achieves perfect recognition even under challenging conditions.

### 4.4. Analysis of Model Convergence

Compared with the convergence of the different models, the number of training epochs required to achieve 100% accuracy was employed as a key metric. Early stopping was used during model training, in which the training process was immediately terminated, once a model consistently achieved 100% accuracy on the validation set over a predefined number of consecutive epochs (referred to as the early stopping threshold). Two key metrics were recorded for analysis and evaluation:

(1) Training epochs: This metric represents the number model iterations that are required from the start of training to 100% accuracy. Fewer epochs indicate a faster convergence, reflecting the model efficiency in learning and adapting to the data.
(2) Training time: This metric represents the total time consumed from the start of training to 100% accuracy. Training time reflects both the model learning speed and its computational efficiency.

**Figure 19:**
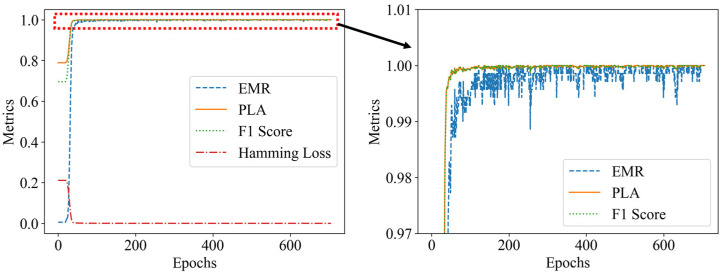
Evaluation metrics of VGG16 with early stopping (early stopping threshold: 10).

**Figure 20:**
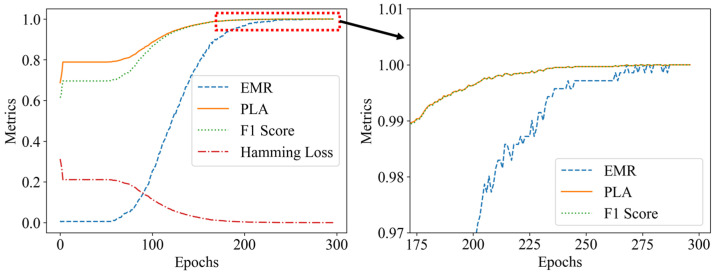
Evaluation metrics of shallow CNN with early stopping (early stopping threshold: 10).

To analyze the training time of each model, early stopping was applied under the condition of applying a maximum load of 15 g and up to two perturbation positions within the 3 × 3 grid. The experiments were conducted on a system equipped with an Intel i9-12900K CPU, 128 GB of DDR5 memory, and an NVIDIA RTX A4000 GPU with 8 GB of VRAM. Each model performance under the different early stopping thresholds is summarized in Table 9, and their accuracy trends are illustrated in Figure 19 and Figure 20.

The results indicate that VGG16 achieved 100% accuracy in the 122nd epoch; however, the EMR oscillated unstably until the 705th epoch. In contrast, the shallow CNN model achieved 100% accuracy in the 268th epoch, and the EMR remained relatively stable, and the model became stabilized in the 296th epoch. The results indicate that the shallow CNN quickly achieves 100% accuracy in the shortest training time. While VGG16 reached a 100% model accuracy relatively early in the training process (by the 122nd epoch). However, VGG16 stabilized at the 705th cycle, which demonstrates that VGG16 exhibits strong specklegram decoupling capability during the early stages as a deep model. However, its performance for specklegram recognition suffers from poor long-term stability and slow convergence. In contrast, the shallow CNN model demonstrates consistent performance, with minimal variation in the number of training epochs required under the different early stopping thresholds. This indicates that this shallow CNN has better stability and convergence in terms of specklegram recognition compared with VGG16 under multiple perturbation locations and intensities. Deep models like VGG16, when trained with occlusion, still exhibit instability due to their inherent bias toward local feature extraction, which conflicts with the global variations of specklegram. Overall, the shallow CNN proposed in this work outperforms the common models such as VGG16 in terms of training efficiency and convergence, making it more suitable for specklegram recognition.

### 4.5. Impact of Occlusion

**Figure 21:**
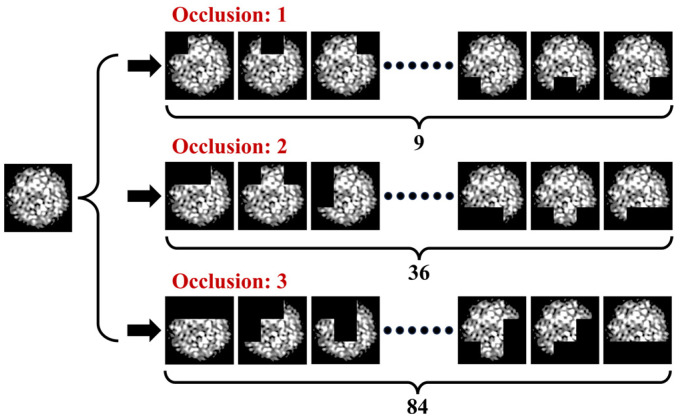
Diagram illustrating specklegram occlusion generation for analyzing the impact of occlusion count on model convergence speed.

The impact of the number of occlusions on the model convergence speed using the early stopping method was analyzed. The dataset used in this analysis was constructed to include only specklegram with no occlusions or a fixed number of occlusions. Specklegrams with mixed occlusion levels were excluded (e.g., the dataset with two occlusions did not include specklegrams with one occlusion), as shown in Figure 21. The training performance of the shallow CNN was tested with two perturbation positions and a maximum of 15 g per position in 3 × 3 grid.

**Figure 22:**
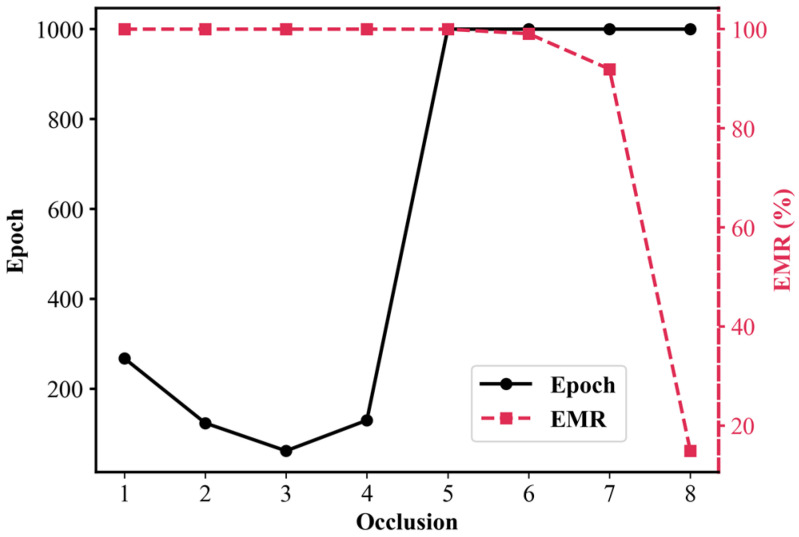
Model convergence and accuracy of the shallow CNN on various datasets with occlusions of 1–8 and early stopping threshold set to 1 (3 × 3 grid: up to 2 perturbation positions with maximum 15 g load per position).

Eight specklegram datasets were constructed with the occlusions from 1 to 8, and the early stopping threshold was set to 1; the performance of the shallow CNN on each dataset is shown in Table 10 and Figure 22. When the occlusion was 1, the model reached 100% EMR at 268 epoch with the fastest training time of 120.59 s. As the occlusion gradually increased to 3, the epoch gradually decreased, which indicates that by randomly discarding the local information of specklegrams, the model’s ability to recognize the global features of specklegrams has been improved and the model convergence also improved. When the occlusion was 4, the model achieved 100% recognition accuracy at 130 training epochs. This indicates that the specklegram discarded too much local information, leaving the remaining regions insufficient to extract global features, thereby reducing the model’s convergence. As the occlusion gradually increased to 5, 6, and 7, the model’s ability to extract global features further declined as more regions were discarded. As a result, some specklegrams were not accurately recognized. When the occlusion was 8, the model’s recognition accuracy dropped significantly due to a single region being available for model training. This means that the model extracted features only from local regions and cannot extract global features at all. These results indicate that the approach effectively enhances the model recognition ability by discarding a small amount of local specklegram information. Further verifying that the overfitting will occur in the excessive learning of local features in specklegrams. By discarding some local features, the model can learn the perturbation features extracted from other regions of specklegrams, thereby improving its learning ability on multi-variable, multi-class, one-shot specklegram datasets.

Furthermore, we compared the impact of different grids of specklegrams on the convergence of the model, with the early stopping threshold set to 1. As shown in Table 11, the results indicate that more grids lead to faster convergence of the model, as the finer areas are occluded. This also validates the theoretical analysis of the sensitivity in speckle regions. In addition, it should be noted that, except for 2 × 2 grids, although the model training time is close, more grids will generate larger datasets, which will result in longer loading times for the datasets.

## 5. Stability of the Sensor

MMFs are susceptible to external environmental disturbances, particularly temperature variations, which can introduce significant environmental noise. If these noise effects are not mitigated, MMF specklegram sensors may lack applicability. To investigate the stability and repeatability of the MMF specklegram-based multi-position loads sensor under varying temperature conditions, ten datasets were collected. These datasets were obtained under the condition of applying a maximum load of 15 g and up to two perturbation positions within the 3 × 3 grid. Each dataset was collected at different time intervals, with varying ambient temperatures ranging from 20.9 °C to 22.4 °C. The ambient temperatures of these datasets are shown in Table 12.

Since each dataset contains only one specklegram for each load form, it corresponds to only a single environmental state. For a one-shot specklegram dataset, the diversity and richness of the data play a crucial role in the environmental stability of the sensor. Several models were trained based on different hybrid datasets. Model 1 takes the specklegrams from Dataset 1 for training. Model 2 is trained with Dataset 1–5. Model 3 is trained with Dataset 1, 3, 5, 7, and 9. Model 4 and Model 5 are based on transfer learning in existing MMF specklegram sensors [12,19,20]. Model 4 is trained with Dataset 1 and randomly transferring 17 images from each of the other datasets. Model 5 is trained with Dataset 1 and randomly transferring 35 images from each of the other datasets. The model performance with a 3 × 3 and 5 × 5 occlusion grid is shown in Table 13.

The experimental results indicate that Model 3, by learning from a broader range (20.9–22.2 °C) of the speckle dataset, exhibits a significant improvement in recognition accuracy compared to Model 1 and Model 2. This suggests that increasing data richness and diversity can enhance model performance. Although there is an improvement, the accuracy of Model 3 remains a low EMR due to the complexity of the multi-point load identification task. Models 4 and 5, based on transfer learning, improve model robustness by learning from a small amount of data from other datasets. Additionally, Model 5 achieves an EMR of over 92.33% by implementing a more extensive grid traversal occlusion, demonstrating that the transfer learning and traversal occlusion effectively mitigates thermal-induced speckle pattern distortions and enhances the environmental adaptability of the MMF specklegram sensor. Additionally, the design of the MMF speckle sensing system should minimize external disturbances (such as vibrations and deformations), which can be mitigated through various packaging methods.

## 6. Conclusions

The construction method of a multi-variable, multi-class, one-shot specklegram dataset is proposed in this study to recognize multiple perturbation positions and its intensity for an MMF-distributed sensor. The main conclusions are as follows:

(1) The specklegram dataset is prone to overfitting due to the limited number of samples and complex labels. To address this, we conducted theoretical analyses and experiments to investigate the response of local specklegram regions to perturbations. Our findings reveal that different regions of the specklegram exhibit various features when perturbations are applied. We propose a data augmentation strategy using specklegram traversal occlusion to enhance global feature recognition and mitigate overfitting.
(2) To further address overfitting, we introduce a shallow CNN architecture that balances complexity and generalization while improving the decoupling of multimode interference fields. Compared to other deep learning models, our shallow CNN achieves state-of-the-art accuracy with minimal training time, making it highly efficient for specklegram recognition. And it has better global feature extraction ability, making model training more stable.
(3) Moreover, several multi-position load experiments were designed, where random load magnitudes were applied at the different positions along the MMF. The experimental results demonstrated that several neural network models with the proposed method achieved nearly 100% accuracy in recognizing up to 1545 perturbation forms on a planar surface.
(4) The stability of the MMF specklegram-based multi-position load sensor under temperature variations was investigated. Experimental results demonstrated that transfer learning and traversal occlusion improves the recognition accuracy of specklegram-based multi-position load recognition, achieving a recognition accuracy of over 92.33%. The findings highlight the effectiveness of the proposed method in mitigating environmental noise and improving sensor reliability in complex interference environments.

The proposed method provides a new pathway for the advancing of distributed sensing technology using an MMF specklegram sensor. However, an excellent sample collection system needs to be proposed to quickly obtain high-precision and complex perturbation datasets. At the same time, it is necessary to overcome the accuracy degradation caused by speckle distortion generated by environmental noise. In order to improve the anti-interference ability of MMF-distributed sensors against environmental interference, multiple sets of specklegram datasets can be collected to train models under different environmental noises. Future work will focus on high-precision load applications and resistance to environmental interference.

Meanwhile, with the further expansion of the dataset size, this method has the potential to achieve a higher resolution of recognition, laying a solid foundation for the MMF specklegram-distributed sensors. The method proposed here provides a low-cost fiber optic distributed sensing solutions, which can provide technical applications for structural monitoring, aerospace, robotics, and other fields.

## Figures and Tables

**Table 1:** The multi-position sensing capability of proposed and existing sensors.

Reference	Multi-Position Sensing Capability	Perturbation Forms	Revolution of Positioning	Accuracy	Multi-Parameter Recognition	Temperature Compensation
Wei [12]	10-point positioning	10	60 cm	92.83%	No (position only)	Transfer learning
Cuevas [18]	3-point and 10-point positioning	3 and 10	120 cm	99% and 71%	No (position only)	Multi-temperature learning
Ding [19]	9-point positioning	9	0.5 mm	98%	No (position only)	Multi-temperature learning
Sun [20]	4-direction displacement	40	-	97%	Yes (4 parameters)	Not implemented
Fujiwara [21]	3-point with 3-angle bending	25	0.2 cm	-	Yes (3 parameters)	Correlation analysis
Lu [22]	3-point with 3-angle bending	27	20 cm	93.5%	Yes (3 parameters)	Not implemented
proposed	6-point with 3 random loads and 9-point with 2 random loads	1545 and 1351	5 cm	96.63% and 95.78%	Yes (9 parameters)	Transfer learning

**Table 2:** Load combinations of grids.

Grid	Maximum Perturbation Positions	Max Weights	Categories
1 × 2	2	10	121
2 × 2	4	5	1296
2 × 3	3	4	1545
	6	2	729
3 × 3	2	6	1351
	3	2	835
	9	1	512

**Table 3:** Performance of shallow CNN (without occlusion).

Grid	Maximum Perturbation Positions	Max Weights per Cell	EMR	F1	Hamming Loss
1 × 2	2	10	8.00%	0.4495	0.5400
2 × 2	4	5	4.25%	0.6553	0.5267
2 × 3	3	4	9.06%	0.6880	0.3031
	6	2	22.60%	0.7635	0.2352
3 × 3	2	6	3.69%	0.7979	0.1812
	3	2	10.18%	0.7724	0.2102
	9	1	24.27%	0.8405	0.1597

**Table 4:** Performance of shallow CNN (with occlusion).

Grid	Maximum Perturbation Positions	Max Weights per Cell	EMR	F1	Hamming Loss
1 × 2	2	10	100%	1	0
2 × 2	4	5	98.33%	0.9985	0.0019
2 × 3	3	4	96.63%	0.9942	0.0058
	6	2	98.77%	0.9977	0.0023
3 × 3	2	6	95.78%	0.9951	0.0048
	3	2	98.08%	0.9977	0.0023
	9	1	99.12%	0.9990	0.0010

**Table 5:** Performance of the different models on 1 × 2 grid (up to 2 perturbation positions with maximum 50 g load per cell).

Method	Model	EMR	F1	Hamming
Without occlusion	VGG16	4.00%	0.3853	0.6000
	ResNet-18	0.00%	0.3401	0.6400
	CNN-Shallow	8.00%	0.4495	0.5400
With occlusion	VGG16	96.69%	0.9829	0.0165
	ResNet-18	100%	1	0
	CNN-Shallow	100%	1	0

**Table 6:** Performance of the different models on 3 × 3 grid (up to 2 perturbation positions with maximum 15 g load per cell).

Method	Model	EMR	F1	Hamming
Without occlusion	VGG16	4.53%	0.6453	0.3350
	ResNet-18	0.97%	0.5065	0.4358
	CNN-Shallow	9.06%	0.6880	0.3031
With occlusion	VGG16	98.25%	0.9978	0.0029
	ResNet-18	98.06%	0.9967	0.0033
	CNN-Shallow	96.63%	0.9942	0.0058

**Table 7:** Performance of the different models on 3 × 3 grid (up to 9 perturbation positions with maximum 5 g load per cell).

Method	Model	EMR	F1	Hamming
Without occlusion	VGG16	0%	0.7569	0.2254
	ResNet-18	0%	0.7093	0.2050
	CNN-Shallow	4.225%	0.7696	0.1753
With occlusion	VGG16	100%	1	0
	ResNet-18	100%	1	0
	CNN-Shallow	100%	1	0

**Table 8:** Performance of the different models on 2 × 3 grid (up to 3 perturbation positions with maximum 20 g load per cell).

Method	Model	EMR	F1	Hamming
Without occlusion	VGG16	9.71%	0.7718	0.2276
	ResNet-18	1.94%	0.6466	0.3528
	CNN-Shallow	24.27%	0.8405	0.1597
With occlusion	VGG16	99.41%	0.9993	0.0007
	ResNet-18	98.93%	0.9988	0.0012
	CNN-Shallow	99.12%	0.9990	0.0010

**Table 9:** Training performance of the different models with early stopping (3 × 3 grid: up to 2 perturbation positions with maximum 15 g load per cell).

Early Stopping Threshold	Model	Epoch	Training Time (s)
10	VGG16	705	40,947.79
	ResNet-18	783	1195.05
	CNN-Shallow	296	134.18
3	VGG16	250	15,460.77
	ResNet-18	765	1170.18
	CNN-Shallow	282	130.28
1	VGG16	122	7147.60
	ResNet-18	641	978.46
	CNN-Shallow	268	121.32

**Table 10:** Performance of the shallow CNN on various datasets with occlusions of 1–8 and an early stopping threshold set to 1 (3 × 3 grid: up to 2 perturbation positions with maximum 15 g load per position).

Occlusions	Epoch	EMR	Training Time (s)
1	268	100%	120.59
2	124	100%	198.37
3	62	100%	229.57
4	130	100%	686.22
5	1000	99.98%	5374.29
6	1000	99.08%	3583.20
7	1000	91.93%	1548.42
8	1000	14.91%	440.12

**Table 11:** Performance of the difference grids and early stopping threshold set to 1 (up to 2 perturbation positions with maximum 15 g load per position).

Grids of Specklegram	Epoch	EMR	Training Time (s)	Dataset Loading Time (s)
2 × 2	1000	91.19%	242.89	15.82
3 × 3	268	100%	120.59	49.23
4 × 4	163	100%	125.85	55.19
5 × 5	87	100%	105.04	83.68
6 × 6	76	100%	128.78	119.58
7 × 7	53	100%	122.18	161.13

**Table 12:** Specklegram datasets under different temperatures (3 × 3 grid: up to 2 perturbation positions with maximum 15 g load per position).

Dataset	Temperature/°C
1	20.9
2	21.1
3	21.3
4	21.3
5	21.2
6	22.4
7	22.2
8	21.9
9	21.7
10	21.5

**Table 13:** Recognition EMR of Models 1-5 for each dataset (3 × 3 grid: up to 2 perturbation positions with maximum 15 g load per position).

Occlusion Grid	Model	EMR of Dataset									
		1	2	3	4	5	6	7	8	9	10
3 × 3	1	100%	3.12%	0.85%	0.28%	0.00%	0.28%	0.57%	0.28%	0.28%	0.57%
	2	100%	100%	100%	100%	100%	0.57%	0.28%	0.00%	0.57%	0.00%
	3	100%	14.77%	100%	8.81%	100%	4.26%	100%	16.76%	100%	8.81%
	4	100%	46.02%	47.73%	42.33%	38.92%	37.50%	45.74%	43.18%	43.18%	40.34%
	5	100%	68.18%	69.32%	67.61%	62.50%	63.64%	68.47%	69.32%	69.89%	67.61%
5 × 5	1	100%	3.69%	0.85%	0.28%	0.28%	0.28%	0.57%	0.28%	0.28%	0.85%
	2	100%	100%	100%	100%	100%	0.57%	0.57%	0.28%	0.57%	0.00%
	3	100%	10.23%	100%	8.81%	100%	5.40%	100%	16.48%	100%	9.38%
	4	100%	79.83%	78.12%	76.14%	76.99%	71.88%	80.68%	80.11%	79.55%	74.43%
	5	100%	95.17%	92.33%	93.47%	94.89%	93.18%	96.31%	94.60%	96.31%	93.18%

## Data Availability

The data that support the findings of this study are available on request from the corresponding author.
